# Impacts of ovarian reserve on conservative treatment for endometrial cancer and atypical hyperplasia

**DOI:** 10.3389/fendo.2023.1286724

**Published:** 2024-01-05

**Authors:** Pengfei Wu, Weiwei Shan, Yu Xue, Lulu Wang, Sijia Liu, Xiaojun Chen, Xuezhen Luo

**Affiliations:** ^1^ Department of Gynecology, Obstetrics and Gynecology Hospital of Fudan University, Shanghai, China; ^2^ Shanghai Key Laboratory of Female Reproductive Endocrine Related Diseases, Obstetrics and Gynecology Hospital of Fudan University, Shanghai, China

**Keywords:** ovarian reserve, endometrial carcinoma, atypical hyperplasia, endometrial intraepithelial neoplasia, conservative treatment

## Abstract

**Objectives:**

Real-world data indicated that some endometrial atypical hyperplasia (EAH) and early endometrial carcinoma (EEC) patients of fertility preservation had a normal ovarian reserve, while some had a decreased ovarian reserve (DOR). This study was designed to investigate the effect of baseline ovarian reserve on the treatment of EAH and EEC patients who ask for preservation of fertility.

**Methods:**

This was a prospective cohort study conducted at a single university-affiliated fertility center. A total of 102 EAH and EEC patients who received fertility-preserving treatment between March 2019 and August 2020 were included and divided into a DOR group (n=22) and a non-DOR group (n=80).

**Results:**

The 32-week CR rate of the non-DOR group was significantly higher than that of the DOR group (60.3% vs. 33.3%, *P* =0.028). The DOR group had a longer treatment duration to achieve CR than the non-DOR group (40.07 vs. 29.71 weeks, *P*=0.008, HR: 0.54, 95% CI: 0.36–0.86). Multivariate logistic regression analyses demonstrated that DOR (OR: 0.35, 95% CI: 0.13–0.99, *P*=0.049) and BMI ≥25 kg/m^2^ (OR: 0.40, 95% CI: 0.17–0.92, *P*=0.031) were negatively associated with 32-week CR.

**Conclusions:**

Decreased baseline ovarian reserve is negatively correlated with the efficacy of fertility-preserving treatment in EAH and EEC patients, as this group has a lower CR rate and a longer treatment duration to achieve CR than those without DOR.

## 1 Introduction

Endometrial carcinoma is the fifth most common cancer in women (1), and the rate of progression of endometrial atypical hyperplasia (EAH) to cancer is nearly 25% (2). The incidences of endometrial carcinoma and EAH have increased among women of reproductive age in recent decades due to delayed childbearing and the rise in obesity (3). Consequently, progestin fertility-preserving treatment is particularly important for early endometrial carcinoma (EEC) and EAH patients. However, the complete response (CR) rate is 70-80%, and the pregnancy rate is approximately 40% among EEC and EAH patients who ask for fertility preservation (4–8). Thus, studies exploring factors that affect oncofertility outcomes and the administration of individualized treatment according to these risk factors are urgently needed.

Prior research into the efficacy of fertility-preserving treatment suggested that insulin resistance (IR) and obesity are risk factors for the achievement of CR in EAH and EEC (9). In our practice, we found that some EAH and EEC patients had a normal ovarian reserve, while some had a decreased ovarian reserve (DOR). Patients with poor ovarian reserve have lower estrogen level theoretically (10). Elevated levels of estrogen lead to upregulation of estrogen receptor which drives up progesterone receptor expression and the antiproliferative effects of progestin are mediated by the progesterone receptor (11–13). Above all, we hypothesized that poor ovarian reserve might lead to downregulation of progesterone receptor expression and influence treatment efficacy in patients seeking fertility preservation. Nevertheless, to our knowledge, no studies have evaluated the clinical implications of ovarian reserve during progestin treatment in EAH and EEC patients of fertility preservation.

This prospective cohort study was designed to investigate the impacts of baseline ovarian reserve on the onco-fertility outcomes of EAH and EEC patients and to analyze the change in ovarian reserve during fertility-preserving treatment. The results of this study might improve our ability to determine who will benefit from fertility-preserving therapies and to increase the CR rate of fertility-preserving treatment.

## 2 Materials and methods

### 2.1 Study design and population

This prospective, monocentric cohort study was conducted at the Obstetrics and Gynecology Hospital of Fudan University between March 2019 and August 2020. All participants enrolled consecutively in this study and underwent standardized evaluation and fertility-preserving treatment in our center.

To minimize the effect of different regimens on the variation in ovarian reserve and treatment efficacy, patients who received megestrol acetate (MA, 160 mg per day) and/or metformin (MET, 500 mg, thrice daily) were included in this study. The inclusion and exclusion criteria for this study were established according to the National Comprehensive Cancer Network guidelines on fertility-sparing treatment (14). Other inclusion criteria were as follows: (1) first histologically proven EAH or well-differentiated EEC G1 without myometrial invasion; (2) patient age younger than 45 years; and (3) strong willingness to preserve fertility. To minimize the potential bias caused by progestin treatment period on treatment efficacy, patients who took progestins for more than one month before primary comprehensive evaluation in our center were excluded from this study.

All patients were pathologically diagnosed by endometrial biopsy through dilation and curettage with or without hysteroscopy. Another hysteroscopy was performed within 1 month after the initial pathological diagnosis if the patient was diagnosed by dilation and curettage without hysteroscopy. Pathologic diagnosis was confirmed by two experienced gynecological pathologists according to the World Health Organization pathological classification (2014). If their opinions differed, a seminar was held in the pathology department to determine the final diagnosis.

The study was approved by the Ethics Committee of Obstetrics and Gynecology Hospital of Fudan University (ID: 2019-117). Written informed consent was obtained from all enrolled patients for medical treatment and inclusion in this study before initiating treatment.

### 2.2 Conservative treatment protocol

Fertility-preserving treatment was initiated as soon as comprehensive evaluation was completed, and a multidisciplinary team determined whether the patient was suitable for fertility-preserving treatment. Ovarian reserve was considered in decisions of fertility-preserving treatment, but poor ovarian reserve was not a contraindication for fertility preservation. Risks and disadvantages of poor ovarian reserve were fully informed before starting fertility preservation. Therapeutic regimens were decided by doctors. Patients in this study received oral MA (160 mg per day) with or without MET (500 mg, thrice daily). We did not prohibit the use of metformin and we recorded regimens of each patient. A comprehensive hysteroscopic evaluation was performed every 3 months during treatment to evaluate therapeutic efficacy. Endometrial lesions were removed under hysteroscopy, and an endometrial biopsy was randomly performed if no obvious lesion was found. The response to conservative treatment was assessed histologically after each hysteroscopic evaluation. CR was defined as no hyperplasia or cancerous lesion. Partial response (PR) was defined as pathological improvement. Stable disease (SD) was defined as persistence of the initially diagnosed lesion. Progressive disease (PD) was defined as evidence of EEC in patients with EAH or evidence of more severe pathological findings, myometrial invasion, or extrauterine metastasis in EEC patients.

Once a patient achieved CR, the same regimen was continued for another 2–3 months for consolidation. Hysteroscopy was performed 3 months after the first CR for confirmation. The duration to achieve CR was calculated from the time of treatment initiation to the time of first pathological CR if no hyperplasia or cancerous lesion was found in two consecutive hysteroscopic evaluations.

All patients desiring fertility were encouraged to receive assisted reproductive treatments such as *in vitro* fertilization after CR. Low-dose progestin, oral contraceptive pills or the LNG-IUS were used to prevent recurrences in patients who did not plan to become parents. Patients were followed-up every 3 to 6 months after CR. Ultrasound evaluation was performed at each follow-up, and an endometrial biopsy using Pipelle was performed every 6 months during follow-up.

Hysterectomy was strongly recommended for patients with SD for 6 months, PR for 9 months or PD at any time during treatment. For patients who refused hysterectomy, alternative treatments were given according to a multidisciplinary consensus.

### 2.3 Data collection

The general information of the patients, including age, weight, height, basic blood pressure and comorbidities (e.g., hypertension or diabetes), was collected before any treatment was given. Body mass index (BMI) was calculated as weight (kg)/height^2^ (m^2^), and BMI ≥ 25 kg/m^2^ was considered overweight (15).

Blood samples were collected before initiating fertility-preserving treatment, and fasting blood glucose, fasting insulin, lipid profiles and sex hormone profiles were evaluated. AMH was tested at baseline and each follow-up for pathological evaluation (every hysteroscopy or Pipelle evaluation). All samples were collected and examined in the laboratory of the Obstetrics and Gynecology Hospital as previously described (9). All blood samples for determination were obtained under the same preanalytical conditions (sample collection, handling and storage). Plasma samples were assayed for AMH using an iFlash Immunoassay Analyzer (Immunotech, Shenzhen YHLO Biotech Co., Ltd., Shenzhen, China) according to the manufacturer’s protocol. The sensitivity of the assay was 0.01 ng/mL. The intra- and interassay variabilities were 5% and 8%, respectively. The homeostasis model assessment-insulin resistance (HOMA-IR) index (fasting blood glucose [mmol/l] × fasting insulin [microU/ml]/22.5) was used to evaluate IR status (16). Patients with a HOMA-IR index ≥2.95 were defined as insulin resistant (17). The diagnostic criteria for diabetes mellitus, metabolic syndrome and hypertension have been previously described (9).

Because the menstrual cycle is irregular in most EAH and EEC patients, we assessed ovarian reserve by measuring only the anti-Müllerian hormone (AMH) concentration, not the antral follicle count or day-3 follicle-stimulating hormone level. We defined DOR as AMH<1.1 ng/ml and non-DOR as AMH≥ 1.1 ng/ml (18–21).

### 2.4 Statistical analysis

All descriptive data are presented as the mean and SD for data with a Gaussian distribution and as the median plus range or interquartile range for non-Gaussian distributed data. Categorical variables are presented as frequencies with percentages. Continuous variables were analyzed using Student’s t-test or the Mann–Whitney U test, as appropriate. The chi-square test was used to analyze categorical variables except if the expected frequency was <5; in these cases, Fisher’s exact test was used. The Kaplan–Meier method was used to estimate the therapeutic duration; differences between groups were tested using the log-rank test. Adjusted odds ratios (ORs) and 95% confidence intervals (95% CIs) were estimated with a logistic regression model for analyses of the relationship between covariates and 32-week CR. A paired t test was used to assess the variation in AMH with time. Statistical significance was considered as P<0.05 in two-sided tests. Statistical analyses were performed using SPSS (version 23.0, IBM, Armonk, NY, USA).

## 3 Results

### 3.1 General characteristics of patients

The flowchart of the inclusion of patients in this trial is presented in **Supplementary Figure 1** (22). Among all 308 patients screened, 206 were excluded because they did not meet the inclusion criteria. The reasons for exclusion were as follows: coming to our center after receiving progestin for more than one month before initiating fertility-preserving treatment (n=75, including 53 EAH and 22 EEC), being treated by regimens other than MA or MA+MET (n=98, including 71 EAH and 27 EEC), choosing hysterectomy before the first hysteroscopic evaluation in our center (n=14, including 4 EAH and 10 EEC) and not providing consent (n=19, including 10 EAH and 9 EEC). In total, 102 patients were ultimately analyzed in this study: 22 in the DOR group and 80 in the non-DOR group. One patient (1 EAH in the non-DOR group) was lost to follow-up at the 16th week, and 2 patients (1 EAH in the DOR group and 1 EEC in the non-DOR group) were lost to follow-up at the 32nd week.

The general characteristics of the enrolled patients are presented in **Table 1**. A total of 4/32 (12.5%) EEC patients and 18/70 (25.7%) EAH patients had DOR, and no significant differences were found in the proportion of DOR patients between different pathological diagnoses (*P*=0.132). Patients in the non-DOR group were younger than those in the DOR group (age 30.9 years vs. 35.1 years, *P*=0.020). More patients had MS in the DOR group than in the non-DOR group (50.0% vs. 27.5%, *P*=0.046). In total, 34 patients used metformin for IR, for weight loss or by themselves in this study. There were no differences in pretreatment BMI, serum estradiol level, treatment or other comorbidities between the two groups.

**Table 1:** General characteristics of the study population.

	Ovarian reserve group		Overall	*P* value
	DOR	Non-DOR		
**Patient number, n (%)**	22(21.6%)	80(78.4%)	102	–
**Pathological diagnosis, n (%)**				0.132
**EEC**	4/32 (12.5%)	28/32 (87.5%)	32	–
**EAH**	18/70 (25.7%)	52/70 (74.3%)	70	–
**Age at diagnosis (years)** **Median (IQR)**	35.1 (30.5-37.7)	30.9 (28.3-34.9)	31.3 (28.4-36.0)	**0.020**
**BMI (kg/m^2^)** **Median (IQR)**	26.41 (21.30-29.12)	24.76 (21.90-28.52)	25.23 (21.79-28.66)	0.834
**Estradiol (pg/ml)** **Median (IQR)**	61.0 (26.00-116.50)	53.0 (26.00-99.00)	54.5 (26.25-102.25)	0.623
**Diabetes mellitus, n (%)**	3(13.6%)	8(10.0%)	11(10.8%)	0.699
**Hypertension, n (%)**	4(18.2%)	14(17.5%)	18(17.6%)	0.941
**Insulin resistance, n(%)**	8(36.4%)	39(48.8%)	47(46.1%)	0.302
**Metabolic syndrome, n(%)**	11(50.0%)	22(27.5%)	33(32.4%)	**0.046**
**Treatment, n (%)**				0.734
**MA**	14(63.6%)	54(67.5%)	68(66.7%)	–
**MA+MET**	8(36.4%)	26(32.5%)	34(33.3%)	–
**Median treatment duration to CR (range) (weeks)**	40.07 (14.29-102.29)	29.71 (10.86-84.86)	30.29 (10.86-102.29)	**0.008**
**CR, n (%)**				–
**16-week CR**	1/22(4.5%)	15/79(19.0%)	16/101(15.8%)	0.183
**32-week CR**	7/21(33.3%)	47/78(60.3%)	54/99(54.5%)	**0.028**
**Median follow-up duration (range) (weeks)**	128.72 (54.29-172.00)	132.50 (36.00-198.71)	132.00 (36.00-198.71)	–
**Pregnancy**^**a**^**, n (%)**	3/11(27.3%)	35/53(66.0%)	38/64(59.4%)	**0.039**
**Live-birth**^**a**^**, n (%)**	1/11(9.1%)	20/53(37.7%)	21/64(32.8%)	0.085

DOR, decreased ovarian reserve; EEC, early endometrial carcinoma; EAH, endometrial atypical hyperplasia; IQR, interquartile range; BMI, body mass index, BMI = weight/height^2^; MA, megestrol acetate, 160 mg/day; MET, metformin, 1500 mg/day; CR, complete response.

a Among patients who plan for parenthood.

The bold values denote statistical significance at P<0.05 level.

### 3.2 Effects of baseline ovarian reserve on fertility-preserving treatment outcome

**Figure 1:**
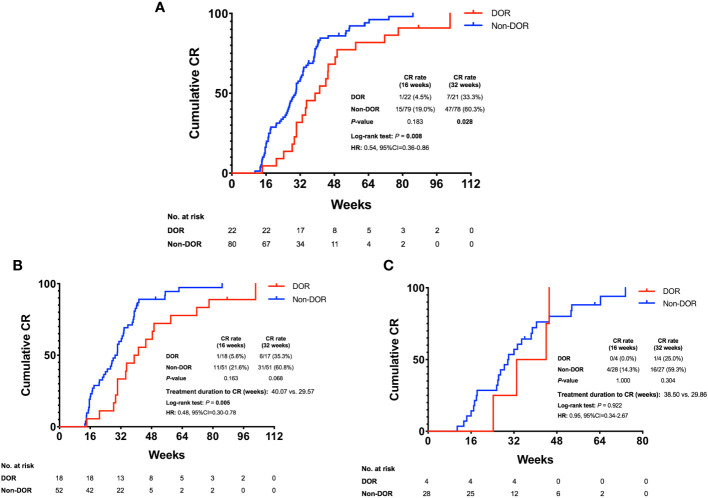
Cumulative CR rate in EAH and EEC patients. **(A)** Cumulative CR rate of all DOR and non-DOR patients. **(B)** Cumulative CR rate in the subgroup of EAH patients. **(C)** Cumulative CR rate in the subgroup of EEC patients. EAH, endometrial atypical hyperplasia; EEC, early endometrial cancer; DOR, decreased ovarian reserve; CR, complete response; HR, hazard ratio; 95% CI, 95% confidence interval.

All patients were followed-up until December 2022. The median follow-up time from the date of initiating treatment to the last follow-up was 132.00 weeks (range 36.00-198.71 weeks). To investigate the effects of baseline ovarian reserve on fertility-preserving treatment in EAH and EEC patients, we analyzed the CR rate and therapeutic duration to achieve CR in patients with different levels of ovarian reserve (**Table 1**; **Figure 1A**). The 16-week CR rate of the non-DOR group (15/79, 19.0%) was higher than that of the DOR group (1/22, 4.5%), but the difference did not reach significance (*P*=0.183). The 32-week CR rate of the non-DOR group was significantly higher than that of the DOR group (60.3% vs. 33.3%, *P*=0.028). The median therapeutic duration to achieve CR in the DOR group was significantly longer than that in the non-DOR group (40.07 vs. 29.71 weeks, *P*=0.008, hazard ratio (HR): 0.54, 95% CI: 0.36–0.86). Stratified analysis by pathological diagnosis was further performed (**Figures 1B, C**). No differences were found in the 16-week and 32-week CR rates when comparing the DOR group with the non-DOR group for either EAH or EEC patients. Among EAH patients, the median therapeutic duration to achieve CR in the DOR group was significantly longer than that in the non-DOR group (40.07 vs. 29.57 weeks, *P*=0.005, HR: 0.48, 95% CI: 0.30–0.78). However, among EEC patients, the median therapeutic duration to achieve CR was not different between the DOR group and non-DOR group (38.50 vs. 29.86 weeks, *P*=0.922).

**Figure 2:**
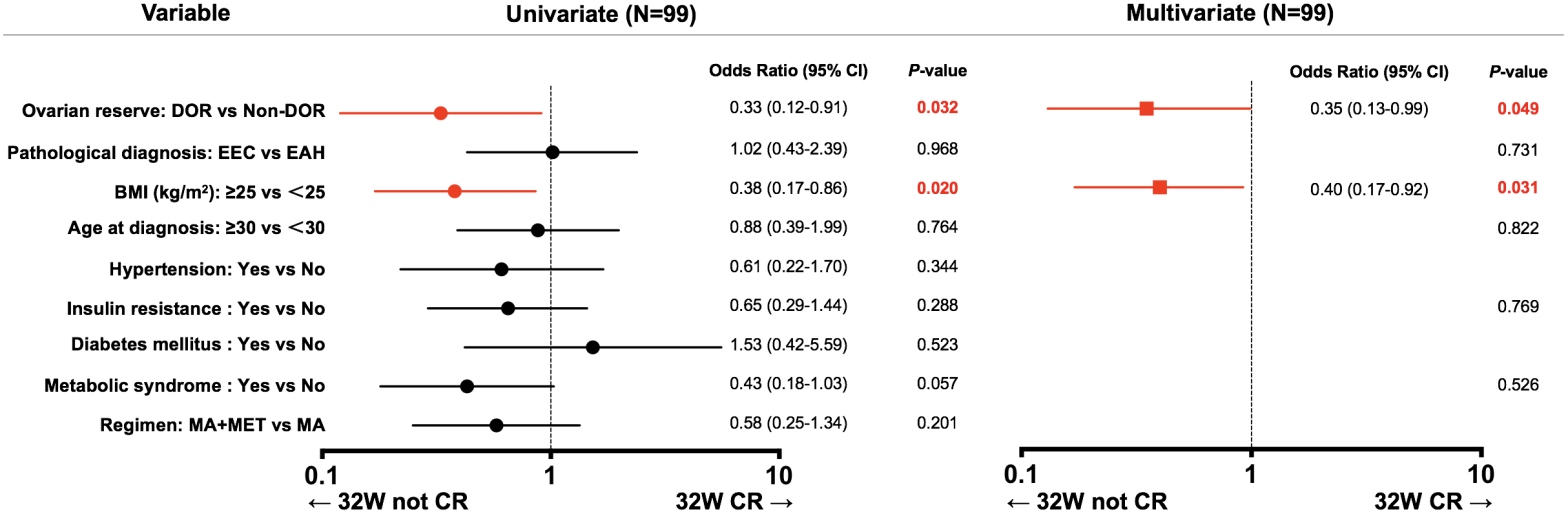
Uni- and multivariate analyses of factors associated with 32-week CR. 95% CI, 95% confidence interval; DOR, decreased ovarian reserve; EAH, endometrial atypical hyperplasia; EEC, early endometrial cancer; BMI, body mass index; MA, megestrol acetate; MET, metformin; CR, complete response.

We then performed univariate and multivariate logistic regression analyses to determine whether different baseline ovarian reserve levels were related to 32-week CR in EAH and EEC patients receiving fertility-preserving treatment (**Figure 2**). Univariate logistic regression analysis showed that DOR (OR: 0.33, 95% CI: 0.12–0.91, *P*=0.032) and BMI ≥25 kg/m^2^ (OR: 0.38, 95% CI: 0.17–0.86, *P*=0.020) were correlated with a lower 32-week CR rate. Multivariate analysis showed that DOR (OR: 0.35, 95% CI: 0.13–0.99, *P*=0.049) and BMI ≥25 kg/m^2^ (OR: 0.40, 95% CI: 0.17–0.92, *P*=0.031) were still correlated with a lower CR rate after adjustment for baseline ovarian reserve, pathological diagnosis, BMI, age, IR and metabolic syndrome. To determine if these parameters are closely correlated, we performed collinearity diagnostics by calculating the variance inflation factor. The variance inflation factors of these parameters were all less than 1.6.

The pregnancy and live-birth rate are also shown in **Table 1**. Among those patients who achieved CR, only 11 women in the DOR group and 53 women in the non-DOR group attempted to conceive. At the time of last follow-up, pregnancy rate was significantly higher in the non-DOR group than in the DOR group (66.0% vs. 27.3%, *P*=0.039). The live birth rate was higher in the non-DOR group, but they did not achieve significant differences (37.7% vs. 9.1%, *P*=0.085).

### 3.3 Variation in ovarian reserve during progestin treatment

**Figure 3:**
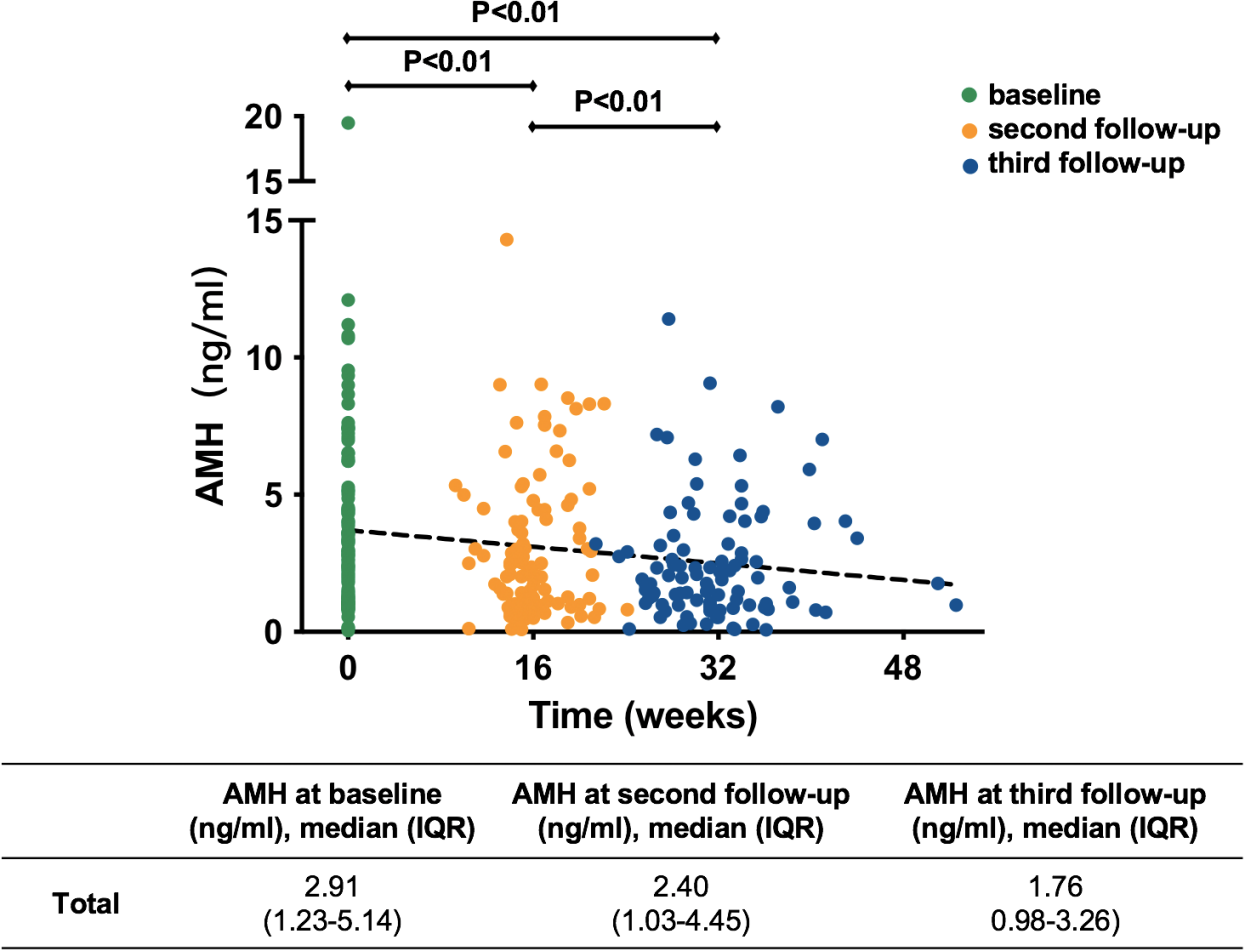
Variation in AMH of all patients during treatment. AMH, anti-Müllerian hormone; IQR, interquartile range.

According to the replacement of therapy after CR in some patients after the third hysteroscopic evaluation, we analyzed only AMH at baseline, before the second hysteroscopic evaluation and before the third hysteroscopic evaluation to observe the change in ovarian reserve during fertility-preserving treatment [**Figure 3**; **Supplementary Figure 2** (22)].

In general, as shown in **Figure 3**, the trend lines indicated that AMH decreased gradually with the extension of treatment time. The median serum AMH concentration was 2.91 ng/ml at baseline, 2.40 ng/ml at the second follow-up and 1.76 ng/ml at the third follow-up. They were all significantly different (*P*<0.01). At the second follow-up during progestin treatment, AMH in 82 (81.2%) patients appeared to decline [**Supplementary Figure 2A** (22)]. The AMH in 32 (31.7%) patients decreased by more than 25% from baseline and that in 6 (5.9%) patients decreased by more than 50% (**Supplementary Figure 2A** [22)]. This trend was more obvious at the third follow-up during progestin treatment [**Supplementary Figure 2B** (22)]. AMH decreased by more than 25% from baseline in 61 (61.6%) patients and by more than 50% from baseline in 30 (30.3%) patients [**Supplementary Figure 2B** (22)].

To exclude the effect of metformin on ovarian reserve, we conducted stratified analyses by the use of metformin [**Supplementary Table 1** (22)]. The results indicated that metformin had no significant effects on the change in ovarian reserve during progestin treatment.

## 4 Discussion

The current prospective study was designed to investigate the relationship between baseline ovarian reserve and fertility-preserving outcomes. In this study, approximately 22% of patients had DOR when they started fertility-preserving treatment. Our study demonstrated that decreased baseline ovarian reserve was correlated with a lower CR rate and longer therapeutic duration to achieve CR than non-DOR.

To date, continuous progestin-based therapy is widely accepted as the main treatment for selected patients who wish to preserve their fertility (14). Previous research suggested that IR and overweight were risk factors in the achievement of CR in EAH and EEC patients (9). To date, the reasons for progestin resistance in endometrial cancer include overexpression of epidermal growth factor receptor (EGFR) and activation of the PI3K/Akt pathway (23–26). To our knowledge, our study is the first to demonstrate DOR as an independent risk factor for achieving CR. Theoretically, women with poor ovarian reserve have lower estrogen levels (10). As previous studies reported, elevated estrogen levels could upregulate estrogen receptors and drive up progesterone receptor expression (11, 12). Moreover, progesterone receptor could mediate the antiproliferative effects of progestin (13). Therefore, we hypothesized that the low concentration of estrogen in patients with DOR might lead to low expression of progesterone receptor and result in low response to progestin treatment. However, no significantly differences were observed between the DOR group (61.0 pg/ml, IQR: 26.00-116.50 pg/ml) and the non-DOR group (53.0 pg/ml, IQR: 26.00-99.00 pg/ml) in baseline estrogen levels (*P*=0.623). The possible reasons for the conflicting results may be as follows: (1) Estrogen level is significantly affected by menstrual cycle phase, but EAH and EEC patients often have irregular menstruation, it is hard to ensure that blood samples are collected at the same menstrual cycle phase. (2) The estrogen level of the endometrium may not be the same as the serum estrogen level, so serum estrogen levels may not reflect actual estrogen levels in the endometrium. Further studies are needed to test this hypothesis.

Several studies have reported achieving improved therapeutic effects by prolonging treatment duration in EAH and EEC patients who asked for preservation treatment (27). However, the efficacy of prolonging the treatment duration was different in the DOR group in our study. The 16-week CR rate was low in both the non-DOR group and the DOR group (19.0% vs. 4.5%, *P*=0.183), but the 32-week CR rate improved differentially in these two groups after the therapeutic time was prolonged. We hypothesized that prolonging the treatment duration could lead to a more severe suppression of ovarian reserve in the DOR group. Patients with decreased baseline ovarian reserve might not benefit from a prolonged therapeutic time. To improve therapeutic efficacy, we probably need to stratify patients according to the baseline ovarian reserve before initiating fertility-preserving treatment.

Serum AMH is a reliable marker of ovarian reserve and is correlated with the size of the primordial follicle pool (10, 28). Our research indicated that AMH declined in DOR and non-DOR patients with prolonged progestin treatment and that the level of AMH decreased more at the third follow-up than at the second follow-up. The hypothalamic–pituitary–ovarian axis is an intricate system that involves positive and negative feedback. MA may cause the inhibition of ovarian reserve regardless of the baseline level. According to this result, we might need to reduce the duration of progestin use and change other protective regimens much earlier when patients achieve CR to decrease the effect of progestin treatment on ovarian reserve. To date, the levonorgestrel intrauterine system (LNG-IUS) and oral high-efficacy progestin are both treatments for EAH and EEC patients who ask for fertility preservation (11). In the literature, the pathological CR rate of patients who received the LNG-IUS was similar to that of patients who received oral progestin (29–31). However, the amount of systemic progestin released by the LNG-IUS was much lower than that of oral progestin, as previously reported (32, 33). Therefore, the patients with low ovarian reserve could probably use the LNG-IUS to reduce the inhibitory effects of progestin treatment. Nevertheless, the decrease in AMH in our study may reflect merely a transient suppression of the ovarian follicles rather than the true loss of ovarian reserve. Future follow-up and studies are needed to demonstrate whether AMH decreases more with the time of progestin treatment or whether ovarian reserve recovers after stopping progestin treatment.

This study has some limitations. First, follow-up time was short, and we could not collect data on the subsequent changes in ovarian reserve patients. Furthermore, we used only the plasma AMH concentration, not the antral follicle count or day-3 follicle-stimulating hormone level, to evaluate ovarian reserve because the menstrual cycle is irregular in most EAH and EEC patients. This may decrease the reliability of the ovarian reserve assessment.

In summary, DOR is negatively correlated with the efficacy of fertility-preserving treatment in EAH and EEC patients, as this group has a lower CR rate and a longer treatment duration to achieve CR than those without DOR. Progestin therapy in fertility-preserving treatment might decrease the ovarian reserve of patients. Further studies are needed to confirm our findings and investigate the mechanisms involved.

## Data availability statement

The original contributions presented in the study are included in the article/**Supplementary Material**. Further inquiries can be directed to the corresponding authors.

## Ethics statement

The studies involving humans were approved by Medical Ethics Committee of Obstetrics and Gynecology Hospital of Fudan University. The studies were conducted in accordance with the local legislation and institutional requirements. The participants provided their written informed consent to participate in this study.

## Author contributions

PW: Data curation, Formal Analysis, Investigation, Software, Writing – original draft. WS: Methodology, Validation, Writing – original draft. YX: Data curation, Software, Writing – review & editing. LW: Data curation, Software, Writing – review & editing. SL: Data curation, Formal Analysis, Writing – review & editing. XC: Conceptualization, Funding acquisition, Project administration, Supervision, Writing – review & editing. XL: Funding acquisition, Project administration, Supervision, Writing – review & editing.

## Supplementary material

Supplementary Figure 1 Flowchart of our prospective study design. EAH, endometrial atypical hyperplasia; EEC, early endometrial cancer; MA, megestrol acetate; MET, metformin; DOR, decreased ovarian reserve; CR, complete response.

Supplementary Figure 2 Variation in AMH from baseline during follow-up. (A) Variation in AMH from baseline to the second follow-up. (B) Variation in AMH from baseline to the third follow-up. AMH, anti-Müllerian hormone.
