# Supplementary material for: Impacts of ovarian reserve on conservative treatment for endometrial cancer and atypical hyperplasia
Source: Front Endocrinol (Lausanne). 2024 Jan 5;14:1286724. doi: 10.3389/fendo.2023.1286724 (PMC10796988; doi:10.3389/fendo.2023.1286724)
Supplement: Supplementary file 1 [file Table_1.docx]

**Supplemental table 1. Variation in AMH in patients receiving different treatments.**

|  | **DOR** | | ***P*-value** | **Non-DOR** | | ***P*-value** | **Overall** | | ***P*-value** |
| --- | --- | --- | --- | --- | --- | --- | --- | --- | --- |
|  | **MA** | **MA+MET** |  | **MA** | **MA+MET** |  | **MA** | **MA+MET** |  |
| **Patient number, n** | 14 | 8 | - | 54 | 26 | - | 68 | 34 | - |
| **AMH at baseline (ng/ml)**  **Median (IQR)** | 0.97  (0.70-1.07) | 0.86  (0.84-1.00) | 0.525 | 3.55  (2.26-6.25) | 4.10  (2.40-7.34) | 0.203 | 2.94  (1.20-4.98) | 2.87  (1.20 -6.29) | 0.347 |
| **Variation of AMH from baseline at the second follow-up (%), median (range)** | -6.52  (-50.00-100.00) | -24.36  (-55.77~ -1.18) | 0.165 | -17.54  (-85.78-40.91) | -15.21  (-58.06-25.23) | 0.483 | -15.74  (-85.78-100.00) | -16.92  (-58.06-25.23) | 0.683 |
| **Variation of AMH after from baseline at the third follow-up (%), median (range)** | -23.21  (-72.22-49.47) | -59.93  (-89.29-15.29) | 0.145 | -30.56  (-80.54-84.28) | -34.73  (-74.83-91.04) | 0.722 | -29.12  (-80.54-84.28) | -37.37  (-89.29-91.04) | 0.263 |

Footnote：

DOR, decreased ovarian reserve; AMH, anti-Müllerian hormone; MA, megestrol acetate, 160 mg/day; MET, metformin, 1500 mg/day; IQR, interquartile range.
