# Supplementary figures and images for: Impacts of ovarian reserve on conservative treatment for endometrial cancer and atypical hyperplasia
Source: Front Endocrinol (Lausanne). 2024 Jan 5;14:1286724. doi: 10.3389/fendo.2023.1286724 (PMC10796988; doi:10.3389/fendo.2023.1286724)

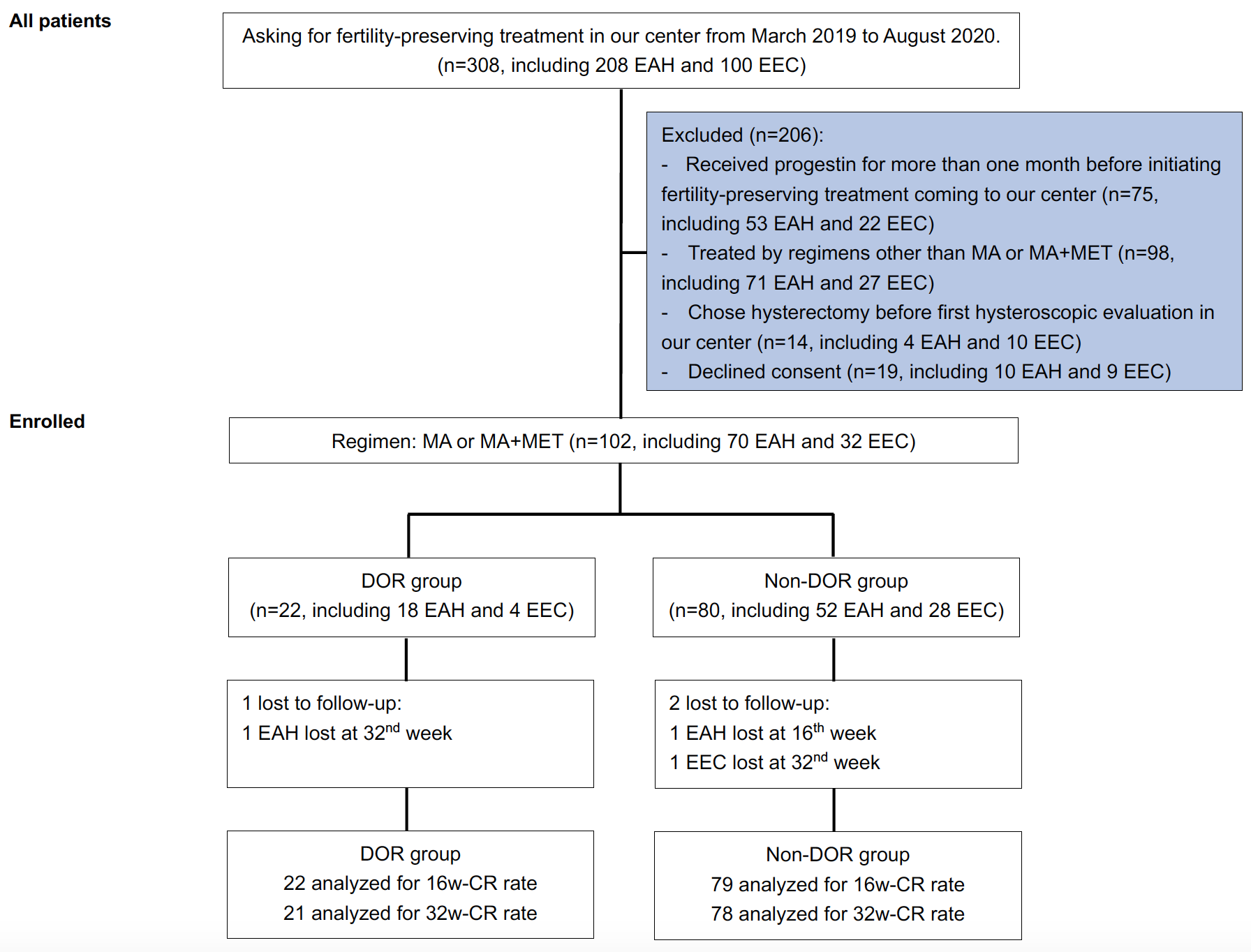

Supplement: Supplementary Figure 1 — Flowchart of our prospective study design. EAH, endometrial atypical hyperplasia; EEC, early endometrial cancer; MA, megestrol acetate; MET, metformin; DOR, decreased ovarian reserve; CR, complete response. [file Image_1.tif]

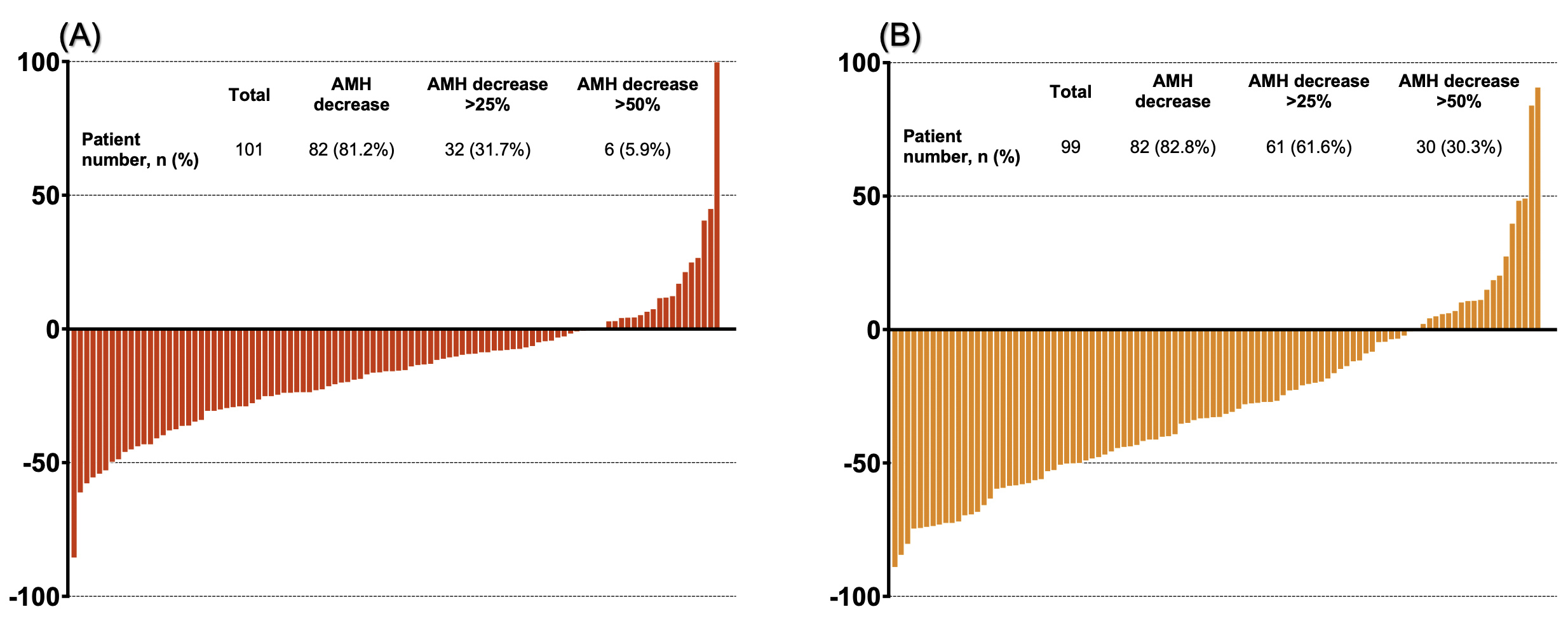

Supplement: Supplementary Figure 2 — Variation in AMH from baseline during follow-up. (A) Variation in AMH from baseline to the second follow-up. (B) Variation in AMH from baseline to the third follow-up. AMH, anti-Müllerian hormone. [file Image_2.tiff]
